# Secretoneurin as a novel predictor of metabolic syndrome in patients with obstructive sleep apnea syndrome

**DOI:** 10.1016/j.bjorl.2026.101856

**Published:** 2026-06-16

**Authors:** İbrahim Arslan, Yasin Aslan, Muzaffer Arı, Hamit Yaşar Ellidağ, Ömer Tarık Selçuk

**Affiliations:** a Antalya Education and Research Hospital, Department of Ear, Nose, and Throat, Antalya, Turkey; b Antalya Education and Research Hospital, Department of Medical Biochemistry, Antalya, Turkey

**Keywords:** Metabolic syndrome, Obstructive sleep apnea, Secretoneurin, Hypoxia, Cardiovascular disease

## Abstract

**Objective:**

This study aimed to evaluate whether Secretoneurin (SN) was elevated in Metabolic Syndrome (MetS) and could serve as a potential predictive biomarker in the development of MetS in patients with Obstructive Sleep Apnea Syndrome (OSAS).

**Methods:**

This cross-sectional study included 86 consecutive participants. Following anthropometric measurements, blood tests, polysomnography, and blood pressure assessments, the participants were categorized into three groups: the MetS(+) OSAS group, the MetS(−) OSAS group, and the control group without MetS or OSAS. Serum SN levels were measured using the enzyme-linked immunosorbent assay method.

**Results:**

The SN levels (pmol/L) of the MetS(+) OSAS group were statistically higher than those of the MetS(−) OSAS group and the control group (p < 0.033). A positive and statistically significant correlation was observed between SN levels (pmoL/L) and serum triglyceride levels (mg/dL) (*r* = 0.244, p = 0.024 < 0.05).

**Conclusions:**

This study indicates that SN may have predictive value in the development of MetS in patients with OSAS.

**Level of evidence:**

4.

## Highlights

• Metabolic syndrome is associated with obstructive sleep apnea syndrome.
• Secretoneurin may contribute to the development of metabolic syndrome.
• Secretoneurin level is positively correlated with triglyceride level.

## Introduction

Obstructive Sleep Apnea Syndrome (OSAS) is a chronic disorder characterized by recurrent episodes of pharyngeal obstruction resulting in apnea and hypopnea during sleep.^1^ In addition to being recognized as an independent risk factor for obesity, diabetes, hypertension, and hypercholesterolemia, OSAS also contributes to the development of Cardiovascular Disease (CVD).^2^, ^3^, ^4^, ^5^, ^6^, ^7^, ^8^ Studies have shown that cardiovascular morbidity is higher in patients with OSAS compared to those without this condition.^9^

Metabolic Syndrome (MetS) is a complex disorder associated with hypertension, abdominal obesity, low High-Density Lipoprotein (HDL) levels, elevated Triglycerides (TG), and high blood glucose. In addition to increasing the risk of type 2 diabetes, MetS can lead to atherosclerotic CVD.^10^ Some studies have demonstrated that MetS is six to nine times more prevalent in patients with OSAS compared to the general population.^11^^,^^12^

Although the mechanisms through which OSAS leads to metabolic disorders have not yet been fully elucidated, systemic inflammation plays a key role in the development of the morbidities associated with OSAS.^13^^,^^14^ High-sensitivity C-reactive protein, a strong indicator of systemic inflammation, may serve as a biomarker for both MetS and CVD.^15^^,^^16^ Furthermore, it has been reported that inflammatory markers such as high-sensitivity C-reactive protein, tumor necrosis factor-alpha, and interleukin-6 increase in OSAS and show a positive correlation with excessive daytime sleepiness.^14^^,^^17^^,^^18^ However, some studies evaluating patients with OSAS have not found significant evidence of systemic inflammation.^19^^,^^20^

Many studies have shown that the prevalence of insulin resistance is high in patients with OSAS and is positively correlated with the severity of OSAS.^21^ Besides obesity, OSAS itself may be an independent factor in the pathogenesis of insulin resistance, as intermittent hypoxia has been shown to cause insulin resistance in healthy individuals.^22^

Oxidative stress is defined as an imbalance between the production of reactive oxygen species and the antioxidant systems that counteract them. Several studies have shown that patients with CVD have high levels of oxidative stress and impaired antioxidant status.^23^^,^^24^ Similarly, patients with MetS have high oxidative stress and decreased antioxidant levels compared to controls without MetS.^25^, ^26^, ^27^ Increased oxidative stress can be considered an early event in the development of MetS and may contribute to the progression of the disease. Demirbag et al.^25^ reported that oxidative stress increased with the number of components of MetS. Considering that obesity is a common risk factor for both OSAS and MetS, patients with OSAS commonly present with MetS or its component disorders. Although obesity is a well-known factor in increasing oxidative stress, recurrent upper airway obstruction in patients with OSAS is also thought to cause oxidative stress. However, some studies have reported increased oxidative stress in patients with OSAS, although not all studies have confirmed this association.^28^, ^29^, ^30^, ^31^

Secretoneurin (SN) is a 33-amino-acid polypeptide derived from the enzymatic processing of secretogranin 2 and is secreted by endocrine, neuroendocrine, and neuronal cells.^32^^,^^33^ SN has been found to induce dopamine release in rat brain striatal slices and to promote neurite outgrowth in cerebellar granule cells.^34^^,^^35^ Following cellular hypoxia, SN expression is upregulated in a tissue-specific manner. Following ischemia, elevated SN levels have been reported in rodent brain tissue and human serum, which may be a result of disruption of the blood-brain barrier and subsequent increased serum levels of this protein.^36^ Furthermore, transient hypoxia in the brain significantly increases SN levels in neuronal cells.^37^ In addition to its effects on the nervous system, SN acts as both a potent chemotactic cytokine and an angiogenesis-inducing agent.^38^^,^^39^

Oxidative stress is a significant factor involved in cellular and subcellular processes that lead to inflammation or, compensatorily, fibrosis, hypertrophy, and/or apoptosis. Since SN has regulatory effects on mesenchymal cells, leukocytes, and endothelial cells, it may play a role in the inflammatory response. SN has been shown to regulate transendothelial leukocyte migration, similar to tumor necrosis factor-alpha.^40^ A recent study explored the regulatory effects of immune cell infiltration and signaling pathway blockade on disease progression.^41^ Inflammatory and immune mechanisms may play a role as a bridge between SN and metabolic disorders. SN may serve as a potential regulator of the immune-metabolic crosstalk pathway.

MetS and OSAS are linked through crosstalk between chronic inflammation and metabolic remodeling mechanisms.^42^ Feng et al. illustrated the relationship between chronic inflammation and local tissue remodeling through MUC13 upregulation, involving the MEK1/2 signaling pathway.^43^ SN may similarly influence metabolic abnormalities through inflammatory and signaling pathways.

SN is associated with mortality risk in CVD, particularly heart failure, as well as in severe sepsis and septic shock.^44^, ^45^, ^46^, ^47^, ^48^, ^49^, ^50^ Furthermore, elevated serum SN levels have been demonstrated in adults with hypoxic brain injury and neonates who have developed hypoxic-ischemic brain injury due to perinatal asphyxia.^51^^,^^52^ In contrast, it has also been reported that SN protects skeletal muscle and myocardium against ischemic injury and apoptosis, and SN gene therapy ameliorates hindlimb and myocardial ischemia in apolipoprotein E deficient mice without affecting systemic atherosclerosis, as well as stimulating coronary angiogenesis, improving left ventricular function, and preventing myocardial remodeling in rat models of myocardial infarction.^53^, ^54^, ^55^

There is some evidence that angiogenesis plays a role in disorders associated with MetS, such as insulin resistance, hypertension, dyslipidemia, and obesity.^56^ Fournier et al. reported that levels of chromogranin A, chromogranin B, and SN were higher in patients with diabetic retinopathy than in those with nondiabetic retinopathy.^57^ Schgoer et al. found that SN gene therapy improved blood flow in an ischemia model in mice with type 1 diabetes by enhancing therapeutic neovascularization.^58^

In the light of these studies, SN may serve as a new peptide that connects the nervous, immune and vascular systems. Thus, in the current study, we hypothesized that SN might be a potential predictive biomarker in the development of MetS in patients with OSAS.

## Methods

### Participants

This cross-sectional study included 86 consecutive participants who presented to the sleep laboratory of the Otorhinolaryngology Clinic at Antalya Training and Research Hospital with suspected OSAS between April 2023 and July 2024. Ethical approval for the study was obtained from the ethics committee of the hospital (decision dated April 13, 2023, and numbered 5/23), and the study was conducted in accordance with the tenets of the Declaration of Helsinki after all participants provided informed consent. Demographic data, smoking status, Body Mass Index (BMI), neck circumference, waist circumference, Apnea-Hypopnea Index (AHI), systolic blood pressure, and diastolic blood pressure were recorded for all participants. Fasting blood glucose, HDL cholesterol, and TG levels were obtained from medical records, which included routine blood test results. The inclusion criteria were age >18-years, suspected OSAS, and willingness to participate in the study. The exclusion criteria were a history of CVD, cerebrovascular disease, chronic obstructive pulmonary disease, and neuroendocrine tumors. Each participant underwent overnight polysomnography, after which they were evaluated for MetS. Based on this evaluation, the participants were categorized into three groups: the MetS(+) OSAS group, the MetS(−) OSAS group, and the control group without MetS or OSAS.

### Polysomnography

Overnight polysomnography was performed using a Grass Telefactor PMA AS40 device (AstroNova, Inc., West Warwick, RI, USA) in the sleep laboratory. Scoring was conducted by the same examiner according to the recommendations in the American Academy of Sleep Medicine Manual for the Scoring of Sleep included in the third edition of the International Classification of Sleep Disorders of the same society. The classification criteria were as follows: normal, AHI <5; mild OSAS, AHI 5–15; moderate OSAS, AHI 15–30; and severe OSAS, AHI >30.^59^

### Definition of MetS

The diagnosis of MetS was based on the criteria updated in 2005 by the American Heart Association/National Heart, Lung, and Blood Institute within the framework of the National Cholesterol Education Program Adult Treatment Panel-III. According to these criteria, the presence of at least three of the following five conditions was accepted as MetS^60^:

1 Waist circumference ≥102 cm for men or ≥88 cm for women.
2 Serum TG levels ≥150 mg/dL or use of medication for hypertriglyceridemia.
3 Serum HDL cholesterol levels <40 mg/dL for men or <50 mg/dL for women or use of medication for low HDL.
4 Systolic blood pressure ≥130 mmHg or diastolic blood pressure ≥85 mmHg or use of antihypertensive medication for hypertension.
5 Fasting plasma glucose levels ≥100 mg/dL or use of medication for hyperglycemia.

### Measurement of SN levels

A 5 mL peripheral venous blood sample was collected from each participant after overnight fasting. The samples were centrifuged at 3,000 rpm for 10-minutes, and the serum portion was separated and stored at −80 °C. Serum SN levels were measured using the enzyme-linked immunosorbent assay method following the manufacturer’s protocol (ELISA Kit, BT LAB, Shanghai, China) in the biochemistry laboratory of our hospital.

### Statistical analysis

Descriptive statistics were presented as frequency, percentage, mean, standard deviation, median, minimum, maximum, 25th percentile (Q1), and 75th percentile (Q3) values. For the analysis of categorical data, the Pearson Chi-Square test was used since the percentage of cells with expected values less than 5 was below 20%. Statistically significant differences between column proportions were evaluated using the *z*-test and Bonferroni correction for multiple comparisons. The normality assumption was assessed using the Shapiro-Wilk test. For numerical data comparisons among the three groups, one-way analysis of variance was applied when the data followed a normal distribution. Post hoc pairwise comparisons were undertaken using the Tukey test for significant results. For non-normally distributed data, the non-parametric Kruskal-Wallis H test was used, and pairwise comparisons were conducted using the Bonferroni-Dunn procedure for significant results. Statistical analyses were performed using SPSS version 23.0, and a p-value of <0.05 was considered statistically significant. We used the formula suggested by Feng et al.^61^ to calculate the required respondent sample size. Accordingly, we found the estimated sample size to be 40, and our sample size was consistent with the estimated sample size.

## Results

Among the 86 patients included in the study, 45.3% were in the MetS(+) OSAS group, 39.5% were in the MetS(−) OSAS group, and 15.1% were controls. Of all patients, 32.6% were female and 67.4% were male. Table 1 presents the comparison of groups based on general characteristics. There were statistically significant differences in age among the groups (p = 0.019 < 0.05), with the MetS(+) OSAS group having a significantly higher median age compared to the remaining groups. Differences in BMI values were also statistically significant (p < 0.0001), with the MetS(+) OSAS group exhibiting the highest BMI values. For neck and waist circumference, statistically significant differences were observed across groups (p = 0.001 < 0.05 and p < 0.0001, respectively), with the MetS(+) OSAS group displaying higher values compared to the other groups. There was a statistically significant difference in sex ratios between the groups (p = 0.027 < 0.05). In the MetS(−) OSAS group, 20.6% of the patients were female, whereas the proportion of females in the control group was 61.5%, with a statistically significant difference between these proportions. The proportion of female patients in the MetS(+) OSAS group was 33.3%, which was not statistically different from either of the remaining two groups. Similar results were observed for the proportion of male ratios. While 79.4% of the patients in the MetS(−) OSAS group were male, the proportion of male patients in the control group was 38.5%, indicating a statistically significant difference. In addition, male patients constituted 66.7% of those in the MetS(+) OSAS group, which did not differ statistically from the other two groups. There were no statistically significant differences in smoking rates across the groups (p = 0.99 > 0.05).

**Table 1:** Comparison of groups based on general characteristics.

	MetS(+) OSAS group	MetS(−) OSAS group	Control group	Total	p-value
	n (%)	n (%)	n (%)	n (%)	
Sex					
Female	13 (33.3)^c^	7 (20.6)^b^	8 (61.5)^a^	28 (32.6)	0.027^1^
Male	26 (66.7)^c^	27 (79.4)^b^	5 (38.5)^a^	58 (67.4)	
Total	39 (100)	34 (100)	13 (100)	86 (100)	
Smoking status					
Smoker	27 (69.2)^a^	24 (70.6)^a^	9 (69.2)^a^	60 (69.8)	0.99^1^
Non-smoker	12 (30.8)^a^	10 (29.4)^a^	4 (30.8)^a^	26 (30.2)	
Total	39 (100)	34 (100)	13 (100)	86 (100)	

	Median (Q_1_–Q_3_)	Median (Q_1_–Q_3_)	Median (Q_1_–Q_3_)		
Age	50 (45–57)^a^	42.5 (38–52)^b^	45 (40–50)^b^		0.019^1^
BMI (kg/m^2^)	33.3 (31.3–38.5)^a^	29.35 (27.5–32)^b^	25 (24.2–28)^b^		<0.0001^1^
Neck circumference (cm)	40 (38–44)^a^	39 (36–41)^b^	37 (36–39)^b^		0.001^1^
Waist circumference (cm)	110 (104–118)^a^	102 (93–110)^b^	97 (90–104)^b^		<0.0001^2^

^1^Pearson Chi-Square test, ^2^One-way analysis of variance test. Different lowercase letters within the same row indicate statistically significant differences between column values (p < 0.05). MetS, Metabolic Syndrome; OSAS, Obstructive Sleep Apnea Syndrome; BMI, Body Mass Index.

Table 2 presents the comparison of OSAS severity rates between groups, revealing statistically significant differences (p = 0.005 < 0.05). Mild OSAS was observed in 10.3% of the patients in the MetS(+) OSAS group and in 41.2% of those in the MetS(−) OSAS group, revealing a statistically significant difference. Moderate OSAS was present in 23.1% of the patients in the MetS(+) OSAS group and in 23.5% of those in the MetS(−) OSAS group, with no statistically significant difference. Severe OSAS was observed in 66.7% of the MetS(+) OSAS cases and in 35.3% of the MetS(−) OSAS cases, showing a statistically significant difference.

**Table 2:** Comparison of OSAS severity between the OSAS groups.

	MetS(+) OSAS group	MetS(−) OSAS group	Total	p-value*
	n (%)	n (%)	n (%)	
OSAS severity				
Mild	4 (10.3)^a^	14 (41.2)^b^	18 (24.7)	0.005
Moderate	9 (23.1)^a^	8 (23.5)^a^	17 (23.3)	
Severe	26 (66.7)^a^	12 (35.3)^b^	38 (52.1)	
Total	39 (100)	34 (100)	73 (100)	

*Pearson Chi-Square test. Different lowercase letters within the same row indicate statistically differences between column values (p < 0.05). MetS, Metabolic Syndrome; OSAS, Obstructive Sleep Apnea Syndrome.

Table 3 highlights statistically significant differences among the groups in laboratory and sleep parameters. The MetS(+) OSAS group exhibited the highest AHI values (p < 0.0001) and TG levels (p < 0.0001), with no significant difference in TG levels between the MetS(−) OSAS and control groups. Systolic blood pressure was significantly higher in the MetS(+) OSAS group compared to the others (p < 0.0001), while diastolic blood pressure showed no significant variation (p = 0.112). Similarly, fasting blood glucose levels were elevated in the MetS(+) OSAS group compared to the remaining groups (p = 0.016), but no significant differences were noted between the MetS(−) OSAS and control groups. No significant group differences were observed for HDL levels (p = 0.094). In the comparison of sleep latency (min), rapid eye movement latency (min), and sleep efficiency (%) among the groups, there were no statistically significant differences (p = 0.507 > 0.05, p = 0.922 > 0.05, and p = 0.520 > 0.05, respectively). Table 3 also summarizes significant differences among the groups for lowest saturation, desaturation index, desaturation percentage, and awake oxygen saturation. The control group showed the highest lowest saturation (%) compared to the MetS(+) OSAS and MetS(−) OSAS groups (p < 0.0001), with no difference between the latter two groups. The desaturation index and desaturation percentage also differed significantly across all groups (p < 0.0001), with the MetS(+) OSAS group exhibiting the highest values and the control group the lowest. Awake oxygen saturation was significantly lower in the MetS(+) OSAS group compared to the other groups (p < 0.0001), with no difference between the MetS(−) OSAS and control groups.

**Table 3:** Comparison of groups based on laboratory and sleep parameters.

	MetS(+) OSAS group (n = 39)	MetS(−) OSAS group (n = 34)	Control group (n = 13)	
	Median (Q_1_–Q_3_)	Median (Q_1_–Q_3_)	Median (Q_1_–Q_3_)	p-value*
AHI	35.9 (25.1–62.1)^a^	20.7 (8.1–38.2)^b^	3.4 (2.4–3.9)^c^	<0.0001
Serum TG level (mg/dL)	175 (146–213)^a^	105.5 (82–131)^b^	90 (76–122)^b^	<0.0001
Serum HDL level (mg/dL)	47 (37–57)	52 (47–57)	50 (46–56)	0.094
Systolic blood pressure (mmHg)	120 (120–130)^a^	120 (110–120)^b^	110 (110–120)^b^	<0.0001
Diastolic blood pressure (mmHg)	75 (70–80)	70 (70–80)	70 (70–75)	0.112
Fasting blood glucose (mg/dL)	100 (92–117)^a^	91.5 (85–98)^b^	92 (86–94)^c^	0.016
Sleep latency (min)	6.5 (2–16.5)	3.75 (1.5–11.5)	8.5 (2.5–15)	0.507
REM latency (min)	104.5 (66–164.5)	93.5 (72–168.5)	92.5 (73.5–134.5)	0.922
Sleep efficiency (%)	90.4 (83.6–94.3)	93.5 (86–95.6)	88.8 (82.6–96.2)	0.52
Lowest saturation (%)	81 (75–86)^b^	84 (80–87)^b^	91 (90–93)^a^	<0.0001
Desaturation index	32.7 (21.9–53.1)^a^	18.55 (7.3–31.2)^b^	3.7 (2.4–4.3)^c^	<0.0001
Desaturation percentage (%)	10.1 (2.6–26)^a^	3.15 (1.1–9.6)^b^	0 (0–0)^c^	<0.0001
Awake oxygen saturation (%)	94.3 (93.7–95.2)^b^	95.85 (94.9–96.4)^a^	96.4 (95.6–97.2)^a^	<0.0001

*Kruskal–Wallis H test. Different lowercase letters within the same row indicate statistically significant differences between column values (p < 0.05). MetS, Metabolic Syndrome; OSAS, Obstructive Sleep Apnea Syndrome; AHI, Apnea-Hypopnea Index; TG, Triglycerides, REM, Rapid Eye Movement.

**Fig. 1:**
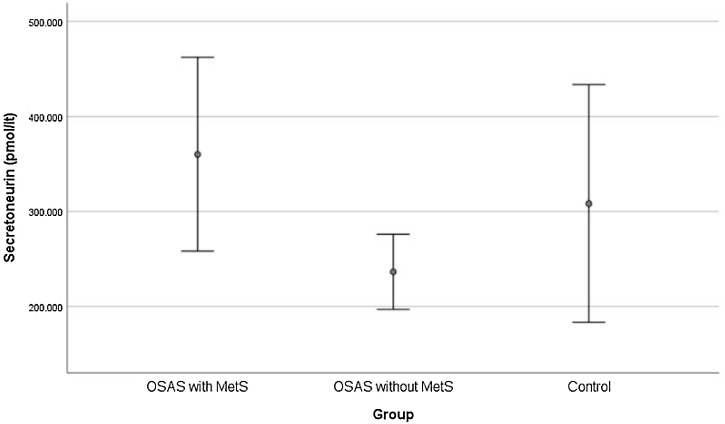
Comparison of secretoneurin (pmol/L) levels across groups.

The SN levels (pmol/L) of the MetS(+) OSAS group were statistically significantly higher than those of the MetS(−) OSAS and control groups. Although the SN levels were higher in the MetS(−) OSAS group than in the control group, this difference was not statistically significant (Fig. 1).

Table 4 shows the relationship between SN levels (pmol/L) and MetS parameters. Among these variables, only the SN level (pmol/L) and the serum TG level (mg/dL) were found to be statistically significantly and positively correlated (*r* = 0.244, p = 0.024 < 0.05).

**Table 4:** Correlation between the secretoneurin level (pmol/l) and clinical parameters.

Secretoneurin level (pmol/L)	Age	BMI (kg/m^2^)	NC (cm)	WC (cm)	AHI	Serum TG level (mg/dL)	Serum HDL level (mg/dL)	FBG (mg/dL)	SBP (mmHg)	DBP (mmHg)
*r*	−0.012	0.109	0.195	0.162	0.103	0.244*	−0.145	0.088	0.030	0.087
p	0.911	0.319	0.072	0.137	0.344	0.024	0.181	0.420	0.786	0.428
n	86	86	86	86	86	86	86	86	86	86

*Statistically significant at the 0.05 level. SN, Secretoneurin; WC, Waist Circumference; TG, Triglycerides; HDL, High-Density Lipoprotein; FBG, Fasting Blood Glucose; SBP, Systolic Blood Pressure; DBP, Diastolic Blood Pressure; MetS, Metabolic Syndrome; AHI, Apnea-Hypopnea Index; NC, Neck Circumference; BMI, Body Mass Index; *r*, Spearman correlation coefficient.

## Discussion

In the current study, we found that serum SN levels were significantly higher in the MetS(+) OSAS group compared to both the MetS(−) OSAS and control groups. Among the polysomnography parameters, AHI, desaturation percentage, and desaturation index were significantly elevated in the MetS(+) OSAS group, while awake oxygen saturation was significantly lower in this group. These findings suggest that MetS, which could be considered a complication of OSAS, is associated with the severity of OSAS, and this association is consistent with the literature.^62^

It has been found that the prevalence of MetS decreases with age, and cardiometabolic comorbidities associated with OSAS decrease accordingly.^63^ Several prospective studies have shown that the association between OSAS and hypertension, coronary heart disease, heart failure, and mortality is strongest in the youngest, and OSAS is not a significant risk factor for adverse health outcomes in older men and women.^64^^,^^65^ In one study, the highest prevalence based on AHI and clinical criteria was around the age of 55 for men and around the age of 65 for women. The same study also revealed that oxygen desaturation was significantly less in older individuals, suggesting that OSAS is more severe in young and middle-aged individuals.^66^ In our study, the median age was 50-years among patients with MetS(+) OSAS, while it was 42.5-years among those with MetS(−) OSAS.

MetS is common in patients with OSAS, and obesity is a shared risk factor. On the other hand, based on previous literature, it is not certain whether the overlapping metabolic dysfunction between OSA and MetS is solely a consequence of underlying obesity. Increasing evidence suggests that insulin resistance develops in patients with OSAS, independent of overall adiposity. Tissue hypoxia triggers insulin resistance and its clinical consequences in patients with OSAS, regardless of BMI.^67^ Additionally, MetS has been shown to be associated with OSAS when the AHI cut-off value is 10 or higher and with increasing severity of AHI.^68^ We found that the AHI values of patients with OSAS who also had MetS ranged from 25.1 to 62.1.

In this study, interestingly, the SN levels of the MetS(−) OSAS group were found to be lower than those of the control group, although this difference was not statistically significant. It was thought that this finding might be due to metabolic changes caused by subclinical features that may be associated with OSAS in control participants who presented with suspected OSAS.

Studies have demonstrated that TG levels increase in OSAS and that the severity of nocturnal hypoxia correlates positively with TG levels.^69^, ^70^, ^71^ A number of meta-analyses have been published demonstrating associations between TG levels and CVD risk, independent of the HDL level.^72^^,^^73^ Ma et al. found that TG levels were positively correlated with insulin resistance in individuals with normal glucose tolerance, while they were negatively correlated with beta cell function in those with dyslipidemia. Therefore, the authors suggested that hypertriglyceridemia control should be strengthened in individuals with dyslipidemia, even when the glucose tolerance test is normal.^74^ In our study, not only were TG levels in the MetS(+) OSAS group significantly higher than those in the MetS(−) OSAS and control groups, but we also found a weak positive correlation between SN levels and TG levels. These findings suggest that SN may have an effect on TG metabolism, and this effect may contribute to the development of MetS in patients with OSAS.

This study has certain limitations that should be acknowledged. First, we did not use the Epworth Sleepiness Scale and therefore could not assess excessive daytime sleepiness in participants. Second, some patients had a history of medication use for hyperglycemia, hypertension, and dyslipidemia. Excluding medications that directly affect the metabolic and inflammatory profile in MetS might have provided a more accurate reflection of SN levels. Third, because of the cross-sectional descriptive design, the study cannot establish a cause-effect relationship between SN and MetS. Fourth, we could not perform polysomnography on healthy individuals for ethical reasons; thus, the control group consisted of individuals who presented to the clinic with suspected OSAS who had an AHI of <5 according to polysomnography but no MetS, which resulted in a smaller sample size. Finally, since patients with MetS but without OSAS were not evaluated, it is not known whether serum SN levels are affected by MetS independently of OSAS.

## ORCID ID

Yasin Aslan: 0000-0002-9775-6028

Muzaffer Arı: 0000-0002-9639-538X

Hamit Yaşar Ellidağ: 0000-0002-7511-2547

Ömer Tarık Selçuk: 0000-0002-7364-7599

## Conclusions

In conclusion, this study indicates that SN may have a predictive value in the development of MetS in patients with OSAS.

## Funding

No funding was received for this work. We confirm that we have given due consideration to the protection of intellectual property associated with this work and that there are no impediments to publication, including the timing of publication, with respect to intellectual property.

## Data availability statement

The authors declare that all data are available in repository.

## Declaration of competing interest

The authors declare no conflicts of interest related to this study. No financial, personal, or professional relationships influenced the research, authorship, or publication of this manuscript. All authors have read and approved the manuscript.

## References

[1] Malhotra A., White D.P. (2002). Obstructive sleep apnoea. Lancet..

[2] Lavie L. (2003). Obstructive sleep apnoea syndrome--an oxidative stress disorder. Sleep Med Rev..

[3] Lira A.B., de Sousa Rodrigues C.F. (2016). Evaluation of oxidative stress markers in obstructive sleep apnea syndrome and additional antioxidant therapy: a review article. Sleep Breath..

[4] Mohit, Tomar M.S., Sharma D., Nandan S., Pateriya A., Shrivastava A. (2023). Emerging role of metabolomics for biomarker discovery in obstructive sleep apnea. Sleep Breath..

[5] Young T., Palta M., Dempsey J., Skatrud J., Weber S., Badr S. (1993). The occurrence of sleep-disordered breathing among middle-aged adults. N Engl J Med..

[6] Nieto F.J., Young T.B., Lind B.K. (2000). Association of sleep-disordered breathing, sleep apnea, and hypertension in a large community-based Study. Sleep Heart Health Study. JAMA..

[7] Li J., Thorne L.N., Punjabi N.M. (2005). Intermittent hypoxia induces hyperlipidemia in lean mice. Circ Res..

[8] Peppard P.E., Young T., Palta M., Skatrud J. (2000). Prospective study of the association between sleep-disordered breathing and hypertension. N Engl J Med..

[9] Gozal D., Kheirandish-Gozal L. (2008). Cardiovascular morbidity in obstructive sleep apnea: oxidative stress, inflammation, and much more. Am J Respir Crit Care Med..

[10] Alberti K.G., Eckel R.H., Grundy S.M. (2009). Harmonizing the metabolic syndrome: A joint interim statement of the International Diabetes Federation Task Force on Epidemiology and Prevention; National Heart, Lung, and Blood Institute; American Heart Association; World Heart Federation; International Atherosclerosis Society; and International Association for the Study of Obesity. Circulation..

[11] Coughlin S.R., Mawdsley L., Mugarza J.A., Calverley P.M., Wilding J.P. (2004). Obstructive sleep apnoea is independently associated with an increased prevalence of metabolic syndrome. Eur Heart J..

[12] Gruber A., Horwood F., Sithole J., Ali N.J., Idris I. (2006). Obstructive sleep apnoea is independently associated with the metabolic syndrome but not insulin resistance state. Cardiovasc Diabetol..

[13] Lavie L., Lavie P. (2009). Molecular mechanisms of cardiovascular disease in OSAHS: the oxidative stress link. Eur Respir J..

[14] Ryan S., Taylor C.T., McNicholas W.T. (2009). Systemic inflammation: a key factor in the pathogenesis of cardiovascular complications in obstructive sleep apnoea syndrome?. Thorax..

[15] Kim J., Hakim F., Kheirandish-Gozal L., Gozal D. (2011). Inflammatory pathways in children with insufficient or disordered sleep. Respir Physiol Neurobiol..

[16] Aurora R.N., Punjabi N.M. (2013). Obstructive sleep apnoea and type 2 diabetes mellitus: a bidirectional association. Lancet Respir Med.

[17] Pack A.I., Gislason T. (2009). Obstructive sleep apnea and cardiovascular disease: a perspective and future directions. Prog Cardiovasc Dis..

[18] Testelmans D., Tamisier R., Barone-Rochette G. (2013). Profile of circulating cytokines: impact of OSA, obesity and acute cardiovascular events. Cytokine..

[19] Tam C.S., Wong M., McBain R., Bailey S., Waters K.A. (2006). Inflammatory measures in children with obstructive sleep apnoea. J Paediatr Child Health..

[20] Taheri S., Austin D., Lin L., Nieto F.J., Young T., Mignot E. (2007). Correlates of serum C-Reactive Protein (CRP) ‒ no association with sleep duration or sleep disordered breathing. Sleep..

[21] Lévy P., Bonsignore M.R., Eckel J. (2009). Sleep, sleep-disordered breathing and metabolic consequences. Eur Respir J..

[22] Louis M., Punjabi N.M. (2009). Effects of acute intermittent hypoxia on glucose metabolism in awake healthy volunteers. J Appl Physiol..

[23] Demirbag R., Yilmaz R., Kocyigit A. (2005). Relationship between DNA damage, total antioxidant capacity and coronary artery disease. Mutat Res..

[24] Mallat Z., Philip I., Lebret M., Chatel D., Maclouf J., Tedgui A. (1998). Elevated levels of 8-iso-prostaglandin F2alpha in pericardial fluid of patients with heart failure: a potential role for in vivo oxidant stress in ventricular dilatation and progression to heart failure. Circulation..

[25] Demirbag R., Yilmaz R., Gur M. (2006). DNA damage in metabolic syndrome and its association with antioxidative and oxidative measurements. Int J Clin Pract..

[26] Ford E.S., Mokdad A.H., Giles W.H., Brown D.W. (2003). The metabolic syndrome and antioxidant concentrations: findings from the Third National Health and Nutrition Examination Survey. Diabetes..

[27] Fujita K., Nishizawa H., Funahashi T., Shimomura I., Shimabukuro M. (2006). Systemic oxidative stress is associated with visceral fat accumulation and the metabolic syndrome. Circ J..

[28] Yamauchi M., Nakano H., Maekawa J. (2005). Oxidative stress in obstructive sleep apnea. Chest..

[29] Barceló A., Miralles C., Barbé F., Vila M., Pons S., Agustí A.G. (2000). Abnormal lipid peroxidation in patients with sleep apnoea. Eur Respir J..

[30] Christou K., Moulas A.N., Pastaka C., Gourgoulianis K.I. (2003). Antioxidant capacity in obstructive sleep apnea patients. Sleep Med..

[31] Svatikova A., Wolk R., Lerman L.O. (2005). Oxidative stress in obstructive sleep apnoea. Eur Heart J..

[32] Fischer-Colbrie R., Kirchmair R., Kähler C.M., Wiedermann C.J., Saria A. (2005). Secretoneurin: a new player in angiogenesis and chemotaxis linking nerves, blood vessels and the immune system. Curr Protein Pept Sci..

[33] Helle K.B. (2010). Regulatory peptides from chromogranin A and secretogranin II: putative modulators of cells and tissues involved in inflammatory conditions. Regul Pept..

[34] Saria A., Troger J., Kirchmair R., Fischer-Colbrie R., Hogue-Angeletti R., Winkler H. (1993). Secretoneurin releases dopamine from rat striatal slices: a biological effect of a peptide derived from secretogranin II (chromogranin C). Neuroscience..

[35] Gasser M.C., Berti I., Hauser K.F., Fischer-Colbrie R., Saria A. (2003). Secretoneurin promotes pertussis toxin-sensitive neurite outgrowth in cerebellar granule cells. J Neurochem..

[36] Shyu W.C., Lin S.Z., Chiang M.F. (2008). Secretoneurin promotes neuroprotection and neuronal plasticity via the Jak2/Stat3 pathway in murine models of stroke. J Clin Invest..

[37] Martí E., Ferrer I., Blasi J. (2001). Differential regulation of chromogranin A, chromogranin B and secretoneurin protein expression after transient forebrain ischemia in the gerbil. Acta Neuropathol..

[38] Reinisch N., Kirchmair R., Kähler C.M. (1993). Attraction of human monocytes by the neuropeptide secretoneurin. FEBS Lett..

[39] Kirchmair R., Gander R., Egger M. (2004). The neuropeptide secretoneurin acts as a direct angiogenic cytokine in vitro and in vivo. Circulation..

[40] Kähler C.M., Schratzberger P., Kaufmann G. (2002). Transendothelial migration of leukocytes and signalling mechanisms in response to the neuropeptide secretoneurin. Regul Pept..

[41] Yi M., Li T., Niu M. (2024). Blockade of CCR5+ T cell accumulation in the tumor microenvironment optimizes anti-TGF-β/PD-L1 bispecific antibody. Adv Sci (Weinh)..

[42] Alterki A., Abu-Farha M., Al Shawaf E., Al-Mulla F., Abubaker J. (2023). Investigating the relationship between obstructive sleep apnoea, inflammation and cardio-metabolic diseases. Int J Mol Sci..

[43] Feng C., Zhao Y., Yuan C. (2025). S aureus upregulation of MUC13 modulates mucosal remodeling in chronic rhinosinusitis via MEK1/2 and WNT2B. J Allergy Clin Immunol..

[44] Brynildsen J., Myhre P.L., Lyngbakken M.N. (2019). Circulating secretoneurin concentrations in patients with moderate to severe aortic stenosis. Clin Biochem..

[45] Brynildsen J., Petäjä L., Myhre P.L. (2019). Circulating secretoneurin concentrations after cardiac surgery: data from the FINNish Acute Kidney Injury Heart Study. Crit Care Med..

[46] Myhre P.L., Ottesen A.H., Faaren A.L. (2021). Performance of a novel research-use-only secretoneurin ELISA in patients with suspected acute coronary syndrome: comparison with an established secretoneurin radioimmunoassay. Cardiology..

[47] Ottesen A.H., Carlson C.R., Eken O.S. (2019). Secretoneurin is an endogenous calcium/calmodulin-dependent protein kinase II inhibitor that attenuates Ca^2+^ -dependent arrhythmia. Circ Arrhythm Electrophysiol..

[48] Ottesen A.H., Louch W.E., Carlson C.R. (2015). Secretoneurin is a novel prognostic cardiovascular biomarker associated with cardiomyocyte calcium handling. J Am Coll Cardiol..

[49] Røsjø H., Stridsberg M., Ottesen A.H. (2016). Prognostic value of secretoneurin in critically ill patients with infections. Crit Care Med..

[50] Watanabe T. (2021). The emerging roles of chromogranins and derived polypeptides in atherosclerosis, diabetes, and coronary heart disease. Int J Mol Sci..

[51] Hasslacher J., Lehner G.F., Harler U. (2014). Secretoneurin as a marker for hypoxic brain injury after cardiopulmonary resuscitation. Intensive Care Med..

[52] Wechselberger K., Schmid A., Posod A. (2016). Secretoneurin serum levels in healthy term neonates and neonates with hypoxic-ischaemic encephalopathy. Neonatology..

[53] Røsjø H., Stridsberg M., Florholmen G. (2012). Secretogranin II; a protein increased in the myocardium and circulation in heart failure with cardioprotective properties. PLoS One..

[54] Theurl M., Schgoer W., Albrecht-Schgoer K. (2015). Secretoneurin gene therapy improves hind limb and cardiac ischaemia in Apo E^−^/^−^ mice without influencing systemic atherosclerosis. Cardiovasc Res..

[55] Albrecht-Schgoer K., Schgoer W., Holfeld J. (2012). The angiogenic factor secretoneurin induces coronary angiogenesis in a model of myocardial infarction by stimulation of vascular endothelial growth factor signaling in endothelial cells. Circulation..

[56] Soares R. (2009).

[57] Fournier I., Gaucher D., Chich J.F. (2011). Processing of chromogranins/secretogranin in patients with diabetic retinopathy. Regul Pept..

[58] Schgoer W., Theurl M., Albrecht-Schgoer K. (2013). Secretoneurin gene therapy improves blood flow in an ischemia model in type 1 diabetic mice by enhancing therapeutic neovascularization. PLoS One..

[59] Kapur V.K., Auckley D.H., Chowdhuri S. (2017). Clinical practice guideline for diagnostic testing for adult obstructive Sleep apnea: an American Academy of Sleep Medicine clinical practice guideline. J Clin Sleep Med..

[60] Grundy S.M., Cleeman J.I., Daniels S.R. (2005). Diagnosis and management of the metabolic syndrome: an American Heart Association/National Heart, Lung, and Blood Institute Scientific Statement. Circulation..

[61] Feng C., Yang Y., Chen L. (2022). Prevalence and characteristics of erectile dysfunction in obstructive sleep apnea patients. Front Endocrinol (Lausanne)..

[62] Kumari S., Chaudhary S.C., Sawlani K.K. (2024). Obstructive sleep apnea in metabolic syndrome. Ann Afr Med..

[63] Gaines J., Vgontzas A.N., Fernandez-Mendoza J., Bixler E.O. (2018). Obstructive sleep apnea and the metabolic syndrome: the road to clinically-meaningful phenotyping, improved prognosis, and personalized treatment. Sleep Med Rev..

[64] Lavie P., Lavie L. (2009). Unexpected survival advantage in elderly people with moderate sleep apnoea. J Sleep Res..

[65] Gottlieb D.J., Yenokyan G., Newman A.B. (2010). Prospective study of obstructive sleep apnea and incident coronary heart disease and heart failure: the sleep heart health study. Circulation..

[66] Bixler E.O., Vgontzas A.N., Ten Have T., Tyson K., Kales A. (1998). Effects of age on sleep apnea in men: I. Prevalence and severity. Am J Respir Crit Care Med..

[67] Adeva-Andany M.M., Domínguez-Montero A., Castro-Quintela E., Funcasta-Calderón R., Fernández-Fernández C. (2024). Hypoxia-induced insulin resistance mediates the elevated cardiovascular risk in patients with obstructive sleep apnea: a comprehensive review. Rev Cardiovasc Med..

[68] Parish J.M., Adam T., Facchiano L. (2007). Relationship of metabolic syndrome and obstructive sleep apnea. J Clin Sleep Med..

[69] Drager L.F., Jun J., Polotsky V.Y. (2010). Obstructive sleep apnea and dyslipidemia: implications for atherosclerosis. Curr Opin Endocrinol Diabetes Obes..

[70] Drager L.F., Lopes H.F., Maki-Nunes C. (2010). The impact of obstructive sleep apnea on metabolic and inflammatory markers in consecutive patients with metabolic syndrome. PLoS One..

[71] Newman A.B., Nieto F.J., Guidry U. (2001). Relation of Sleep-disordered breathing to cardiovascular disease risk factors: the Sleep Heart Health Study. Am J Epidemiol..

[72] Stauffer M.E., Weisenfluh L., Morrison A. (2013). Association between triglycerides and cardiovascular events in primary populations: a meta-regression analysis and synthesis of evidence. Vasc Health Risk Manag..

[73] Liu J., Zeng F.F., Liu Z.M., Zhang C.X., Ling W.H., Chen Y.M. (2013). Effects of blood triglycerides on cardiovascular and all-cause mortality: a systematic review and meta-analysis of 61 prospective studies. Lipids Health Dis..

[74] Ma M., Liu H., Yu J. (2020). Triglyceride is independently correlated with insulin resistance and islet beta cell function: a study in population with different glucose and lipids metabolism states. Lipids Health Dis.

